# First report of Edwardsiellosis in cage-cultured sharpsnout sea bream, *Diplodus puntazzo* from the Mediterranean

**DOI:** 10.1186/s12917-015-0482-x

**Published:** 2015-07-21

**Authors:** Pantelis Katharios, Constantina Kokkari, Nancy Dourala, Maria Smyrli

**Affiliations:** Institute of Marine Biology, Biotechnology and Aquaculture, Hellenic Centre for Marine Research, Former American Base of Gournes, Heraklion, 71003 Crete Greece; Selonda Aquaculture, Navarhou Nikodimou 30, 105 56 Athens, Greece

**Keywords:** Edwardsiella, Bacterial disease, Aquaculture, Fish

## Abstract

**Background:**

*Edwardsiella tarda*, is a serious bacterial pathogen affecting a broad range of aquaculture fish species. The bacterium has also been reported as a human pathogen, however recent studies have dissociated the fish pathogenic *Edwardsiella* from those isolated from humans by placing them in a new species, *E. piscicida*. Here we report the first case of Edwardsiellosis in cultured sharpsnout sea breams, *Diplodus puntazzo* in Greece.

**Case presentation:**

The disease has affected cultured sharpsnout sea breams of a commercial fish farm in a single location in East Greece. Two populations of sharpsnout sea breams stocked in two consecutive years in floating cages presented signs of disease which included nodules and abscesses in spleen and kidney, morbidity and cumulative mortality reaching 5.3 %. Using microbiological, biochemical and molecular tools we have identified *Edwardsiella* sp. as the main aetiological factor of the disease. Following phylogenetic analysis the bacterial isolates are grouped with the newly described *Edwardsiella piscicida* species.

**Conclusions:**

This is the first report of Edwardsiellosis in this species but most importantly in sea cage-cultured fish in the Mediterranean which may pose a serious threat for aquaculture fish species in this region.

## Background

*Edwardsiella tarda* is a serious bacterial pathogen affecting a wide range of fish species mostly in warm waters [1]. It is a Gram negative, motile bacterium of the *Enterobacteriacae* family which can be intracellular during infection [2–4] making it less vulnerable to antibiotic treatment [5]. Its importance as a fish pathogen has been gaining increasing interest lately as it is associated with heavy losses in both freshwater and marine cultured fish but also because it can infect humans [6].

The bacterium is responsible for important economic damages in the aquaculture industry of the USA and Asia [5]. In Europe there are few sporadic reports of disease outbreaks in cultured turbot, *Scophthalmus maximus* (L.) mainly from the Atlantic coast in the Gulf of Biscay [7–9] while in the Mediterranean, *E. tarda* has been isolated from diseased feral European eels, *Anguilla anguilla* (L.) from a coastal freshwater lagoon in Spain [10].

Sharpsnout sea bream, *Diplodus puntazzo* (Walbaum) is a well-appreciated sparid which has been considered for many years as one important candidate for the diversification of the Mediterranean aquaculture. Its production however remains low [11] mostly due to the vulnerability of species to various infectious and non-infectious diseases [12–14].

In this communication we report the isolation and partial characterization of *E. tarda* from diseased cultured sharpsnout sea breams. To our knowledge, this is the first isolation of the pathogen in Greece and from this host but more importantly the first report of *E. tarda* from cage-cultured marine fish in the Mediterranean.

## Case presentation

The disease has affected cultured sharpsnout sea breams of a commercial fish farm in a single location in East Greece (Saronikos bay). Two populations of sharpsnout sea breams stocked in two consecutive years into 4 floating cages presented signs of disease. Each cage was stocked with approximately 50–55.000 fish. Two outbreaks of Edwardsiellosis were recorded, the first in October 2013 and the second in July 2014. Table 1 summarizes the production data of the affected stocks and the mortalities observed.

**Table 1:** Production data and mortalities. Production data of the affected sharpsnout sea breams and mortality observed during the two outbreaks of Edwardsiellosis

			First outbreak (October 2013)		Second outbreak (July 2014)	
			Water temperature: 23 °C		Water temperature: 26 °C	
Cage	Date stocked	Age at stocking (months)	Average weight at first outbreak (g)	Cumulative mortality (%)	Average weight at second outbreak (g)	Cumulative mortality (%)
1	April 2012	2	323	4,82	336	4,86
2	April 2012	2	319	5,30	440	5,3
3	July 2012	5	292	4,76	441	4,76
4	April 2013	2	84	2,23	274	2,23

**Fig. 1:**
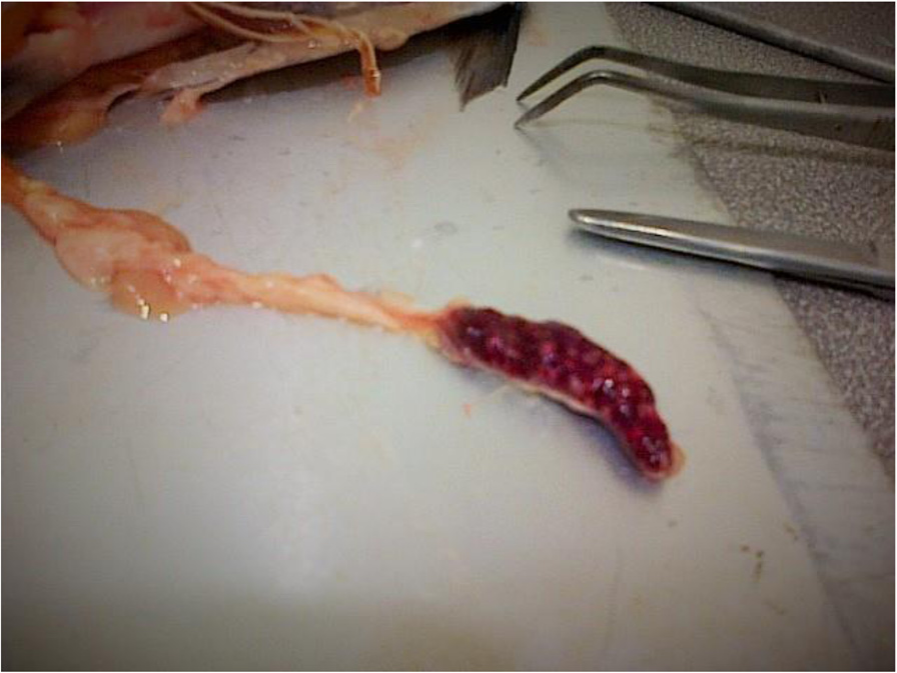
Nodules in spleen. Enlarged spleen of affected fish with multiple whitish nodules

Diseased fish exhibited nodules and abscesses in spleen and kidney (Fig. 1). Pure colonies with identical morphology were obtained at the site by the fish vet of the fish farm in 10 % horse blood Agar (Oxoid) from the kidneys of approximately 30 fish exhibiting signs of distress in each incidence. The initial presumptive diagnosis was performed at the fish farm using API20E bacterial identification kit (BioMerieux, France) on 8–10 pure bacterial colonies from each incidence (*n* = 18). The API profile of all isolates was similar with variability only in the H_2_S production. Other apparently healthy fish species cultured separately in different cages at the same site of the fish farm including gilthead sea bream, *Sparus aurata*, European sea bass, *Dicentrarchus labrax*, meagre, *Argyrosomus regius* and red porgy, *Pagrus pagrus* were also sampled (*n* = 20 per fish species) for bacteriological examination (bacterial cultures in blood agar) during the outbreaks and were negative for *E. tarda*. The fish were treated successfully following oral administration of oxolinic acid in feed (50 mg kg^−1^ biomass for 7 consecutive days).

Pure cultures of two strains (EtS1 and EtS2) isolated from sharpsnout sea bream from each two incidences were sent to the laboratories of HCMR for further characterization. Both isolates formed small distinct colonies. The bacteria were characterized and identified biochemically with BIOLOG GEN III and API20E systems according to manufacturers’ instruction and molecularly using PCR amplifying *Edwardsiella* sp. specific *gyrB* gene coupled with 16 s sequencing [15, 16]. The 16 s rRNA sequence data of the two strains have been deposited in NCBI GenBank under the accession numbers KP729431 and KP729432. In addition, the two strains were screened for the presence of selected virulence genes (*citC, fimA, gadB, katB, mukF* and *esrB*) with PCR [17, 18]. All primers used are presented in Table 2.

**Table 2:** Primers. List of primers used in this study

Gene		Primer’s sequence	Size (bp)	Reference
*gyr*B	F	5′- GCATGGAGACCTTCAGCAAT-3′	415	[15]
	R	5′-GCGGAGATTTTGCTCTTCTT-3′		
*gad*B	F	5′- ATTTGGATTCCCGCTTTGGT-3′	583	[30]
	R	5′- GCACGACGCCGATGGTGTTC-3′		
*muk*F	F	5′- TTGCTGGCTATCGCTACCCT-3′	357	[30]
	R	5′- AACTCATCGCCGCCCTCTTC-3′		
*cit*C	F	5′- TTTCCGTTTGTGAATCAGGTC-3′	596	[30]
	R	5′- AATGTTTCGGCATAGCGTTG-3′		
*fim*A	F	5′- CTGTGAGTGGTCAGGCAAGC-3′	441	[30]
	R	5′- TAACCGTGTTGGCGTAAGAGC-3′		
*esr*B	F	5′-TCGTTGAAGATCATGCCTTGC-3′	311	[30]
	R	5′-TGCTGCGGGCTTTGCTT-3′		
*kat*B	F	5′-CTTAGCCATCAGCCCTTCC-3′	1417	[30]
	R	5′-GCGAGTGCCGTAGTCCTT-3′		
16 s rRNA	27f	5′-AGAGTTTGATCMTGGCTCAG-3′	1465	[16]
	1492r	5′-CGGTTACCTTGTTACGACTT -3′		

The isolates were Gram negative, non-motile, oxidase negative, rods, identified as *E. tarda* with 55 % probability using BIOLOG GENIII. They could utilize several carbon sources such as sugars (maltose, glucose, N-Acetyl-D-glycosamine, N-Acetyl-neuraminic acid, mannose, fructose, glucose-6-PO_4_, fructose-6-PO_4_, serine), aminoacids (galacturonic acid, gluconic acid, glucuronic acid) and caroboxylic acid (lactic acid). Both strains could not utilize arabinose, mannitol and sucrose and exhibited variability in H_2_S production. They could not grow in salinity exceeding 4 % NaCl and they were resistant in acidic pH. They could grow in 20 and 25 °C with best growth at 30 °C.

**Fig. 2:**
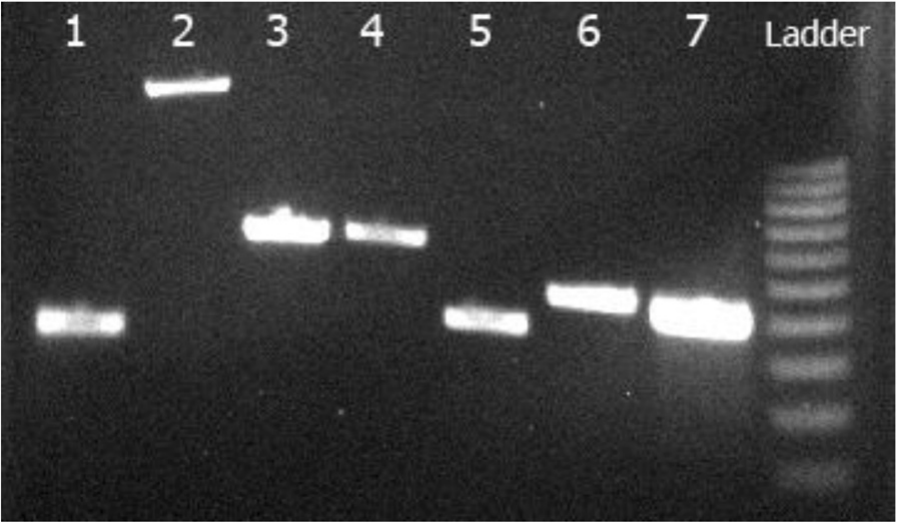
Virulence genes. PCR amplification of selected virulence genes for EtS1. Lanes 1–7: *esr*B, *kat*B, *gad*B, *cit*C, *muk*F, *fim*A, *gyr*B

Identification was further validated after amplification of *gyrB* gene which resulted in a PCR product at the expected size. All virulence genes assessed were present in the isolates (Fig. 2). Haemolysis was tested on horse and sharpsnout sea bream-blood agar. The later was obtained from disease-free broodstock fish of the Institute of Marine Biology, Biotechnology and Aquaculture, HCMR. Strains *E. tarda* (DSM 30052) and *E. hoshinae* (DSM 13771) were used as negative controls and *Aeromonas veronii* strain (Aero1- HCMR pathogen collection) previously shown to be strongly haemolytic on fish blood as positive control. Following 48 h incubation at 25 °C only the *Aeromonas veronii* control strain was positive for haemolysis.

Antibiotic susceptibility of the two isolates was determined with a disk diffusion method [19] on Mueller-Hinton agar for ampicillin (10 μg), oxytetracycline (30 μg), oxolinic acid (2 μg), flumequine (30 μg), florfenicol (30 μg) and sulphamethoxazole/trimethoprim (25 μg) as suggested for fish pathogens [20]. Inhibition diameter was recorded after 24 h incubation at 25 °C. Response was determined according to the breakpoints shown in Table 3.

**Table 3:** Antiobiotic sensitivity. Sensitivity of the two *Edwardsiella* isolates against the antibiotics commonly used in aquaculture (r: radius, S: sensitive, I: intermediate, R: resistant)

	Sensitivity breakpoints (mm)			Radius (mm)		Sensitivity	
Antibiotic	Resistant	Intermediate	Sensitive	EtS1	EtS2	EtS1	EtS2
SFT (25 μg)	r < 14	14 ≤ r ≤ 16	r > 16	16–17	12–13	S	R
OXA (2 μg)	r < 11	11 ≤ r ≤ 13	r > 13	18–19	18–20	S	S
FLO (30 μg)	r < 16	16 ≤ r ≤ 18	r > 18	19–21	20–22	S	S
OTC (30 μg)	r < 15	15 ≤ r ≤ 18	r > 18	15–17	17–18	I	I
FLU (30 μg)	r < 16	16 ≤ r ≤ 19	r > 19	17	22	I	S
AMP (10 μg)	r < 14	14 ≤ r ≤ 18	r > 18	10	12–14	R	R

**Fig. 3:**
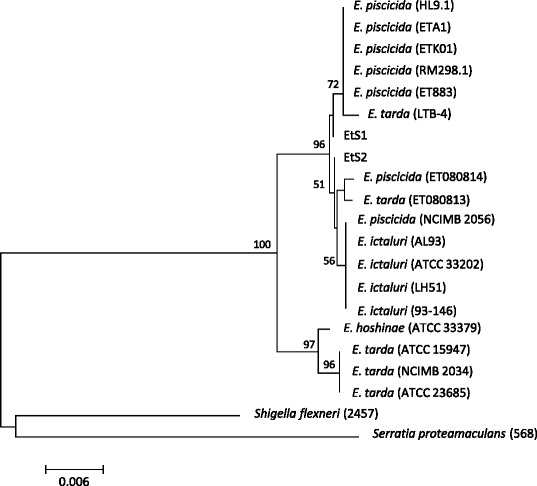
Phylogenetic analysis. Phylogenetic relationships of *Edwardsiella* strains studied as derived from Neighbour-Joining analysis for 16S gene. Numbers on clades indicate the bootstrap values

Phylogenetic relationships of the two isolates were studied using 16 s rRNA gene. The taxonomic status, strain collection numbers and GenBank Accession numbers (http://www.ncbi.nlm.nih.gov/) of all used sequences are presented in Table 4. A total length of 721 bp sequences was used for the phylogenetic analyses. Genetic distances and Neighbor-joining analysis [21] were respectively estimated and performed in MEGA [22] under Tamura-Nei [23] model of evolution. Confidence of tree nodes was tested by bootstrap analyses with 1000 replicates. Both isolates were grouped with *E. ictaluri* and *E. tarda* strains isolated from fish (Fig. 3). Genetic distances between strains EtS1 and EtS2 was 0 %, while the mean genetic distance within the fish-clade was 0.002 % and overall mean distance for the genus was 0.005 %.

**Table 4:** Bacterial strains and codes. Taxonomy, collection codes and accession numbers from NCBI GenBank of the bacterial strains used in this study

Strain	Species	Host/source	Geographic origin	Sequence code
ET883	*E. piscicida*	European eel (*Anguilla anguilla*)	Greaker, Norway (1989)	NR125649
RM 298.1	*E. piscicida*	Turbot (*Scophthalmus maximus*)	Southern Europe (2006)	KC138730
HL 9.1	*E. piscicida*	Turbot (*Scophthalmus maximus*)	Northern Europe (2006)	KC138727
ETA1	*E. piscicida*	Turbot (*Scophthalmus maximus*)	Scotland, UK (2007)	KC138724
ETK01	*E. piscicida*	Korean catfish (*Silurus asotus*)	Jeollabukdo, South Korea (2008)	KC138726
NCIMB 2056	*E. piscicida*	Sea bream (*Evynnis japonicas*)	NCIMB collection	KC138729
LTB4	*E. tarda*	Turbot (*Scophthalmus maximus*)	Qingdao, China (2006)	EU259315
NCIMB 2034	*E. tarda*	Unknown fish spp.	NCIMB collection	KC138728
ET080813	*E. tarda*	Marbled eel (*Anguilla marmorata*)	Qingdao, China (2008)	CP006664
ET080814	*E. tarda*	Japanese eel (*Anguilla japonica*)	Qingdao, China (2008)	KC138723
ATCC 15947^T^	*E. tarda*	Human faeces	Kentucky, USA (1959)	NR024770
ATCC 23685	*E. tarda*	Human faeces		294638553
AL93	*E. ictaluri*	Channel catfish (*Ictalurus punctatus*)	USA	KC138721
ATCC 33202^T^	*E. ictaluri*	Channel catfish (*Ictalurus punctatus*)	Georgia, USA (1976)	NR024769
LH51	*E. ictaluri*	Channel catfish (*Ictalurus punctatus*)	Liaoning, China	EU541494
93–146	*E. ictaluri*	Channel catfish (*Ictalurus punctatus*)	USA	CP001600.2
ATCC 33379^T^	*E. hoshinae*	Female puffin (*Fratercula arctica*)	France	AB682272
EtS1	*Edwardsiella* sp.	Sharpsnout sea bream (*Diplodus puntazzo*)	Greece (2013)	KP729431
EtS2	*Edwardsiella* sp.	Sharpsnout sea bream (*Diplodus puntazzo*)	Greece (2014)	KP729432
2457^T^	*Shigella flexneri*			AE014073
568	*Serratia proteamaculans*			NR074820

## Discussion

This is the first report of Edwardsiellosis in aquaculture fish in Greece and one of the few in the Mediterranean. *Edwardsiella tarda* has been isolated from feral European eels, *Anguilla anguilla* from a coastal freshwater lagoon near Valencia in Spain very close to the Mediterranean Sea [10]. In addition, Edwardsiellosis has also been reported in the Mediterranean coast of Spain in cultured European seabass, *Dicentrarchus labrax*, with the bacterial isolate however resembling *E. ictaluri* [24]. Recently, the assignment of the fish pathogenic isolates to this species has been questioned by Abayneh et al. (2013) [25] who instead proposed a new species, *E. piscicida*. The phylogenetic analysis of the current stains indicate that they are indeed grouped with the strains isolated from other fish. Moreover the biochemical profile of the two isolates is consistent with the profile of *E. piscicida* type strain [25] with the exception of motility. Atypical non-motile virulent *E. tarda* have been isolated from marine fish in Japan [26]. Those strains however were able to utilize arabinose and mannitol unlike the strains described in the current study. Recently, atypical non-motile *E. tarda* strains affecting cultured turbot in China were described [27]. These strains were similar to those described in the current study since besides being non-motile they could also utilize mannose and fructose but not arabinose and mannitol. Whether the strains described herein are *E. piscicida* instead of *E. tarda* needs further investigation since taxonomy of the Edwardsiella genus has not yet been fully resolved. Edwardsiellosis is manifested as a typical acute bacterial septicaemia with external heamorrhages and petechiae in skin and fins and bloody ascites in the peritoneal cavity with liver, spleen and kidney congestions [1, 28]; however it is also associated with more sub-acute lesions such as abscesses and nodules in the visceral organs [8] as in this case. The impact of the disease and its manifestation in terms of external clinical signs may vary depending on fish species. Disease outbreaks occur at high water temperatures [1, 27] usually above 20 °C which is in accordance to the water temperature occurring during the disease outbreaks reported here. Mortality can be severe and is dependent on many different factors such as fish species, age, environmental conditions, handling, stress etc. Host specificity seemed to be an important factor since of the four different species cultured at the specific fish farm only sharpsnout sea bream was affected. The two strains described in this study showed differences in the antiobiotic sensitivity. EtS2 which was isolated at the second incidence was resistant to the potentiated sulfonamide SFT unlike EtS1 which was isolated at the first incidence, while both strains were resistant to ampicillin and sensitive to oxolinic acid and florfenicol. Antibiotic resistance is one of the greatest concerns in aquaculture and *Edwardsiella tarda* isolated from fish has shown that can be multi-drug resistant including resistance to trimethoprime and ampicillin [29]. Virulence of the *Edwardsiella* sp. isolates was not assessed based on *in vivo* experiments due to legal and ethical constraints concerning the use of animals but on their virulent gene repertoire. The current isolates contained the main virulent genes described in literature after thorough functional genomic analysis and screening of virulent and a-virulent *E. tarda* strains [17]. Those genes are implicated in virulence by either providing resistance to the bacterium against host phagocyte killing activity (*GadB, KatB*) [17] indicating also ability for intracellular survival and replication which has been shown for this species [4], or providing the necessary tools for cell adhesion (*fimA*) [17] and penetration (*esrB* which encodes a regulator protein for type III secretion system) [18]. The presence of these genes together with the clinical signs of the disease and the absence of any other pathogen suggest that *Edwardsiella* sp. was the aetiological cause of the disease.

## Conclusions

*Edwardsiella* sp. was isolated from diseased cultured sharpsnout sea bream. It is the first report in this host but also the first report from cultured fish in the Mediterranean. The isolates, identified as *E. tarda* are phylogenetically closer to the newly described *E. piscicida* species. Although confined only to this species until now, the pathogen could potentially spread in other species and pose serious threat to the Mediterranean aquaculture industry.

### Availability of supporting data

The data set supporting the results of this article is included within the article (See Table 4).

## Abbreviations

HCMR: Hellenic Centre for Marine Research
SFT: sulphamethoxazole/trimethoprim
OXA: oxolinic acid
FLO: florfenicol
OTC: oxytetracycline
FLU: flumequine
AMP: ampicillin
